# Supplementary material for: MetaRibo-Seq measures translation in microbiomes
Source: Nat Commun. 2020 Jun 29;11:3268. doi: 10.1038/s41467-020-17081-z (PMC7324362; doi:10.1038/s41467-020-17081-z)
Supplement: Supplementary file 10 — Supplementary Data 7 [file 41467_2020_17081_MOESM10_ESM.zip › File2/Confidence_High_Taxonomy/356724_out.krona.html]

Javascript must be enabled to view this page.

members
magnitude
magnitudeUnassigned
count
unassigned
taxon
rank

356724\_out

104

104
2
superkingdom

74201
phylum
1

1
203494
class

order
48461
1

1
family
1647988

1
genus
239934

1
species

SRS1041142\_contig\_number\_1061
239935

976
phylum
103

class
200643
103

order
171549
103

171550
family
102

239759

SRS014979\_contig\_number\_27420SRS015590\_contig\_number\_contig-100\_1193.111443SRS1041091\_contig\_number\_18481SRS104400\_contig\_number\_37368SRS143070\_contig\_number\_contig-100\_651.90556
102
5
genus

30

SRS013940\_contig\_number\_12081SRS014923\_contig\_number\_42481SRS015960\_contig\_number\_11063SRS016203\_contig\_number\_5993SRS016335\_contig\_number\_17694SRS017821\_contig\_number\_24326SRS018817\_contig\_number\_18352SRS019968\_contig\_number\_4396SRS046369\_contig\_number\_407SRS047433\_contig\_number\_1343SRS048870\_contig\_number\_385SRS050925\_contig\_number\_47SRS053214\_contig\_number\_4031SRS055017\_contig\_number\_26731SRS056259\_contig\_number\_28147SRS063190\_contig\_number\_795SRS065397\_contig\_number\_13723SRS075821\_contig\_number\_1833SRS077730\_contig\_number\_15566SRS1041092\_contig\_number\_341SRS1041132\_contig\_number\_7366SRS1041138\_contig\_number\_7970SRS1041141\_contig\_number\_1053SRS104311\_contig\_number\_12364SRS142890\_contig\_number\_11732SRS143417\_contig\_number\_5010SRS147271\_contig\_number\_11270SRS148511\_contig\_number\_14530SRS893327\_contig\_number\_8324SRS893366\_contig\_number\_27562
species
1872444

1

SRS098571\_contig\_number\_14017
1896969
species

1263036

SRS024625\_contig\_number\_11978SRS064557\_contig\_number\_21335SRS065504\_contig\_number\_38055SRS077231\_contig\_number\_22391SRS1055043\_contig\_number\_8404SRS1055067\_contig\_number\_10404SRS1055099\_contig\_number\_10058
species
7

species

SRS015190\_contig\_number\_14311SRS098881\_contig\_number\_5524
1262694
2

1118061

SRS054352\_contig\_number\_2747
species
1

1946007

SRS144183\_contig\_number\_2312
species
1

55

SRS011084\_contig\_number\_13172SRS012849\_contig\_number\_17939SRS014415\_contig\_number\_1542SRS014534\_contig\_number\_2049SRS014613\_contig\_number\_682SRS014683\_contig\_number\_17728SRS015694\_contig\_number\_17SRS016018\_contig\_number\_329SRS016267\_contig\_number\_10957SRS016954\_contig\_number\_27096SRS019030\_contig\_number\_10829SRS019178\_contig\_number\_351SRS019285\_contig\_number\_5SRS019693\_contig\_number\_2142SRS019787\_contig\_number\_4015SRS021484\_contig\_number\_1754SRS024388\_contig\_number\_11448SRS024492\_contig\_number\_211SRS042628\_contig\_number\_36745SRS043411\_contig\_number\_5778SRS043768\_contig\_number\_30041SRS043841\_contig\_number\_1465SRS044535\_contig\_number\_2127SRS045004\_contig\_number\_4492SRS045645\_contig\_number\_29597SRS046068\_contig\_number\_744SRS051882\_contig\_number\_15006SRS052697\_contig\_number\_21672SRS062654\_contig\_number\_2075SRS063489\_contig\_number\_7538SRS063928\_contig\_number\_527SRS064276\_contig\_number\_38398SRS064757\_contig\_number\_4801SRS075341\_contig\_number\_2921SRS076756\_contig\_number\_2108SRS076804\_contig\_number\_15680SRS077589\_contig\_number\_7863SRS098644\_contig\_number\_45650SRS101376\_contig\_number\_3276SRS1041032\_contig\_number\_8468SRS1041118\_contig\_number\_6100SRS1041145\_contig\_number\_31438SRS1041145\_contig\_number\_38465SRS1055056\_contig\_number\_8826SRS1055069\_contig\_number\_contig-100\_756.96353SRS142503\_contig\_number\_22268SRS142599\_contig\_number\_545SRS143181\_contig\_number\_3758SRS143372\_contig\_number\_2105SRS147557\_contig\_number\_29800SRS148091\_contig\_number\_7680SRS150029\_contig\_number\_3305SRS893341\_contig\_number\_933SRS893373\_contig\_number\_22579SRS971276\_contig\_number\_7819
species
28117

1
171552
family

1
genus
577309

species

SRS077641\_contig\_number\_18628
454155
1
